# A c-MET-Targeted Topical Fluorescent Probe cMBP-ICG Improves Oral Squamous Cell Carcinoma Detection in Humans

**DOI:** 10.1245/s10434-022-12532-x

**Published:** 2022-10-02

**Authors:** Jingbo Wang, Siyi Li, Kun Wang, Ling Zhu, Lin Yang, Yunjing Zhu, Zhen Zhang, Longwei Hu, Yuan Yuan, Qi Fan, Jiliang Ren, Gongxin Yang, Weilong Ding, Xiaoyu Zhou, Junqi Cui, Chunye Zhang, Ying Yuan, Ruimin Huang, Jie Tian, Xiaofeng Tao

**Affiliations:** 1 Department of Radiology, Ninth People’s Hospital, School of Medicine, Shanghai Jiao Tong University, Shanghai, China; 2 Department of Oral and Maxillofacial-Head and Neck Oncology, Ninth People’s Hospital, School of Medicine, Shanghai Jiao Tong University, Shanghai, China; 3 CAS Key Laboratory of Molecular Imaging, Beijing Key Laboratory of Molecular Imaging, The State Key Laboratory of Management and Control for Complex Systems, Institute of Automation, Chinese Academy of Sciences, Beijing, China; 4 Molecular Imaging Center, Shanghai Institute of Materia Medica, Chinese Academy of Sciences, Shanghai, China; 5 Department of Pathology, Ninth People’s Hospital, School of Medicine, Shanghai Jiao Tong University, Shanghai, China

## Abstract

**Introduction:**

The postoperative survival of oral squamous cell carcinoma (SCC) relies on precise detection and complete resection of original tumors. The mucosal extension of the tumor is evaluated visually during surgery, but small and flat foci are difficult to detect. Real-time fluorescence imaging may improve detection of tumor margins.

**Materials and Methods:**

In the current study, a peptide-based near-infrared (NIR) fluorescence dye, c-MET-binding peptide-indocyanine green (cMBP-ICG), which specifically targets tumor via c-MET binding, was synthetized. A prospective pilot clinical trial then was conducted with oral SCC patients and intraoperatively to assess the feasibility of cMBP-ICG used to detect tumors margins. Fluorescence was histologically correlated to determine sensitivity and specificity.

**Results:**

The immunohistochemistry (IHC) results demonstrated increased c-Met expression in oral SCC compared with normal mucosa. Tumor-to-background ratios ranged from 2.71 ± 0.7 to 3.11 ± 1.2 in different concentration groups. From 10 patients with oral SCC, 60 specimens were collected from tumor margins. The sensitivity and specificity of discriminative value derived from cMBP-ICG application in humans were respectively 100% and 75%.

**Conclusions:**

Topical application of cMBP-ICG is feasible and safe for optimizing intraoperative visualization and tumor margin detection in oral SCC patients, which could clinically increase the probability of complete resections and improve oncologic outcomes.

**Supplementary Information:**

The online version contains supplementary material available at 10.1245/s10434-022-12532-x.

Approximately 95% of all head and neck cancers are squamous cell carcinoma (SCC), the most common of which is oral SCC (excluding non-melanoma cutaneous cancers).^1^ Oral SCCs are primarily treated surgically with adequate resection margins to ensure complete resection and to minimize functional impairment related to the procedure. The mucosal extension of the tumor usually is evaluated by visual inspection.^2,3^ However, translation of information from preoperative imaging into the actual situation in the patient during surgery is challenging, and macroscopic evaluation of a tumor in the oral cavity by palpation during surgery is difficult and inaccurate. Moreover, the precursor lesions, such as oral leukoplakia and submucous fibrosis, can subsequently progress to cancers,^4^ whereas the ability to detect malignant transformation early is limited, and preoperative biopsies may suffer from sample error.^5^ Therefore, it is necessary to develop supplementary methods for precise detection of early malignancy and delineation of mucosal extension of the tumor.

The merging of targeted probes with clinical user-friendly imaging technologies provides a powerful and easy tool for the early and precise detection of cancer.^6–8^ Near-infrared (NIR) fluorescence-guided surgery is a promising technique for superficial head and neck lesions using tumor-targeting fluorescent agents (e.g., panitumumab-IRDye800CW, cetuximab-IRDye800CW).^9,10^ For local small-distance metastases, extracellular matrix (ECM) may prevent the penetration of intravenously administered macromolecular agents and cause a higher miss rate for small lesions^11^ due to the different mean fluorescence intensity (MFI) and tumor background ratio (TBR). Instead, mouth-washing topical administration of the targeted probe could be an alternative approach, which could cover the entire oral mucosa and directly screen out suspicious cancer foci without restriction from other tissues.^12^ Meanwhile, the interval between probe administration and imaging would be significantly reduced to minutes and least affected by individual difference.^13,14^

As a c-MET-targeting peptide, c-MET-binding peptide (cMBP) has been developed for tumor imaging and gene delivery.^15–17^ Therefore, instead of intravenous delivery of NIR fluorescence dye, we propose that topically applied, NIR fluorescently labeled, simply synthesized homing-peptide–based, oral SCC-targeting contrast agent, c-MET-binding peptide-indocyanine green (cMBP-ICG), can be preferable for superficial, invasive, and heterogenous tumors such as oral SCC.

This study aimed to determine the safety and feasibility of cMBP-ICG topical application of real-time NIR fluorescence imaging for oral SCC. To study the validity of this method, we conducted a clinical trial for pre- and intraoperative detection of primary tumor and tumor spread.

## Methods

### Synthesis and Characterization of cMBP-ICG

The NIR fluorescent dye, Sulfo-ICG-Maleimide (Adipogen Corporation, San Diego, CA, USA), was conjugated to a custom-synthesized peptide (KSLSRHDHIHHHK) and purified by ChinaPeptides Co. (Suzhou, China) (Fig. S1).

### Clinical Study Design and Participants

An exploratory, open-label, single-arm, single-center, two-group, parallel dose-escalation was held to assess the feasibility of cMBP-ICG for detecting tumor margins and cancer foci. This trial was undertaken with Asian patients and approved by the institutional review board of the Chinese Clinical Trial Registry (ChiCTR2200058058). Written informed consent was obtained from all the patients.

Between October 2021 and February 2022, the study enrolled 10 patients with clinically suspicious, biopsy-proven, primary or recurrent head and neck SCC (details shown in Table S1), who were scheduled to undergo standard-of-care surgery with curative intent at Shanghai Ninth Peoples’ Hospital. All the enrolled patients were 18 years old or older, with a life expectancy longer than 12 weeks. The exclusion criteria for this study and the entire flowchart are shown in Fig. S2.

All the enrolled patients underwent preoperative imaging (CT and/or MRI). The tumor-node-metastasis (TNM) stage was determined in accordance with the American Joint Committee on Cancer (AJCC) eighth edition criteria. According to the time of admission, enrolled patients were classified into two groups. Different concentrations of cMBP-ICG were used for fluorescence imaging in the different groups.

### In Vivo and Ex Vivo Clinical Imaging Procedures

Lyophilized powder of cMBP-ICG (1.0 mg, 3.6 × 10^−4^ mmol) was dissolved in ultrapure water (145.7 or 72.8 mL) to a concentration of 2.5 or 5.0 μM. In order of enrollment, the first five cases used a 5.0-μM concentration for gargling or ex vivo specimen surface smearing, and the next five cases used a 2.5-μM concentration. For preoperative in vivo cMBP-ICG application, the patients gargled 25 mL of cMBP-ICG solution for 30 s, then spit it out (twice). Next, the patients gargled 30 mL of ultrapure water for 30 s and spit it out (twice).

For imaging investigations, videos were acquired before topical application, after topical application pre-wash, and after topical application post-wash using a fluorescence laryngoscope. The camera lens was aimed at the region of interest (ROI) and manually focused. The same instrument settings were used for all the imaging procedures (exposure time, 30 ms; excitation power, 20%; gain, 3 dB, at 60 frames per second). The distance between the lens and surface of the ROI was kept at about 10 mm.^6^ The videos were acquired by slow scanning of the tumor area and surrounding normal mucosa (non-tumor region).

The patients then underwent surgery the next day. For intraoperative ex vivo back-table fluorescence-guided imaging (FGI), the resected tumor specimens were collected directly after excision, and the fresh (non-fixed) samples were incubated with cMBP-ICG at either of the aforementioned concentrations. Then, the samples were rinsed for 1 min with phosphate-buffered saline (PBS) to clear unbound cMBP-ICG (twice), followed by direct NIR fluorescence imaging. Because the fluorescent probe is coated on the surface of resected tumor specimens, the fluorescence images cannot be compared directly with the cross-section. Small tissues from the tumor margin were cut from specimens labeled as high-fluorescence (suspicious of cancer) or low-fluorescence intensity, respectively. The surgeon with 20 years of experience performing maxillofacial surgery, who was blinded to the imaging results, predicted the malignant possibility of specimens according to their visual features and tactile textures. The detection was compared with fluorescence imaging findings, with reference to histopathologic results.

### Histopathologic Processing and Adverse Events Report

Histopathologic processing of the surgical specimens was performed by a board-certified head and neck pathologist. Intraoperatively, frozen-section staining was routinely performed using hematoxylin and eosin (H&E) with real-time guidance from NIR fluorescence imaging. The remaining tumor tissue was embedded in paraffin, and 5-μm-thick sections were obtained. Two neighboring sections were stained with H&E and immunohistochemistry (IHC) for c-MET. A board-certified pathologist masked to NIR fluorescence results evaluated the H&E slides microscopically and outlined the tumor areas. In the analysis of ex vivo tissue, the pathology diagnosis was used as the gold standard for tumor detection.

**Fig. 1:**
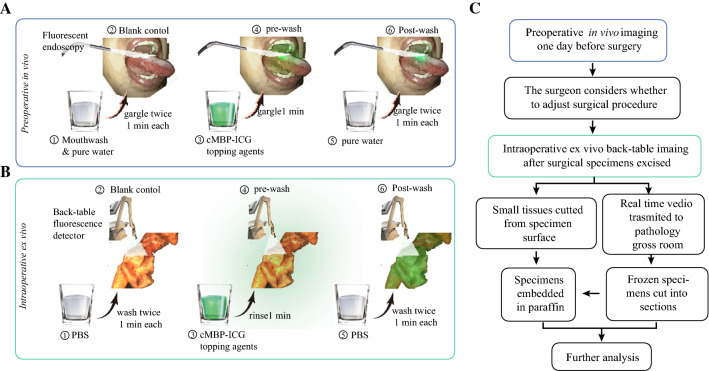
Workflow of fluorescence imaging-based head and neck squamous cell carcinoma (SCC) delineation. **A** After careful mouth-washing (step 1), patients (*n* = 10) with clinically suspicious or biopsy-proven head and neck SCC were examined using a Digital Precision Medicine imaging device H2000 (DPM, Beijing, China) with an endoscopic camera and an ICG-optimized Light Emitting Diode (LED)-filter system. The tumor area and the surrounding margins were imaged as a blank control (step 2). After gargling, a solution of cMBP-ICG (step 3) at different concentrations (2.5 μM or 5 μM, each dose *n* = 5) for 1 min, a pre-wash imaging procedure was performed (step 4). The patients then gargled ultrapure water for 1 min as a clearing solution (step 5), followed by cMBP-ICG administration of post-wash imaging (step 6). **B** Immediately after resection, tissue specimens, including margin samples, were imaged ex vivo in a vertical near-infrared imaging device Z2000 (DPM) following the same protocol described earlier. **C** Preoperative images were taken into consideration during the surgical procedure plan. Intraoperative back-table videos were transmitted in real time to the pathology gross room as frozen section’s references. Small tissues were cut from specimens’ high and low fluorescence intensity surfaces, respectively, for further analysis. cMBP-ICG, c-MET-binding peptide-indocyanine green

Adverse events were reported on the basis of federal regulations and the National Cancer Institute Common Terminology Criteria for Adverse Events (v5.0). The workflow of the clinical trial is shown in Fig. 1.

### Image and Statistical Analysis

For the image analysis, ImageJ software (1.53q, NIH, Bethesda, USA) was used. The tumor-to-background ratio (TBR) was determined as the ratio of fluorescence intensity of tumor ROI (FI_tumor_) and fluorescence intensity of tumor-adjacent tissue ROI (FI_adjacent tissue_). For in vivo imaging, fluorescence intensities of six ROIs were selected from the gross oral SCC tumor area, and six other ROIs were selected from the surrounding non-tumorous tissues. Whenever possible, the ROIs were equally spaced and spread across each area in the surgical field. The mean fluorescence intensity (MFI) was defined as the total fluorescence signal divided by the total number of pixels within the ROI. An overall MFI was calculated using the MFIs from the ROIs. Ex vivo imaging was used to determine the sensitivity and specificity of cMBP-ICG for surgical specimens. A receiver operating characteristic (ROC) analysis was performed on data from the small tissue cut from specimens’ surfaces. The pathologist assessed whether the tumor was present in the tissue using a binary (yes/no) approach according to H&E stains of paraffin sections.

Descriptive statistics, including the MFI and TBR, are presented as mean ± standard deviation. The MFI and TBR of the 10 in vivo lesions and ex vivo specimens from all the patients were compared using the unpaired *t* test (two-tailed). We calculated the predictive values of the fluorescence signal of the fresh tissues against the H&E result. A *P* value lower than 0.05 was considered statistically significant. Statistics and figures were generated using GraphPad Prism (GraphPad, version 8.0, San Diego, CA, USA) and the R language (R Foundation, Vienna, Austria) *pROC* package (version 1.18.0) and the *yardstick* package (version 0.0.9).

## Results

### cMBP-ICG Targets c-MET for Optical Imaging of Human Oral SCC

All 10 patients completed ex vivo fluorescence imaging, and 8 patients completed preoperative in vivo fluorescence imaging. In vivo imaging was not performed for patient 5 or patient 8 due to limitation of mouth opening. Patient 9 was a non-tumor patient, so the MFI for patient 9 was not included in the TBR statistics.

Both preoperative in vivo and intraoperative ex vivo imaging showed the pronounced fluorescence signal in the tumor area compared with the surrounding uninvolved mucosa for both coating concentrations. As the concentration doubled, the mean fluorescence intensity of both the tumor and the background increased. The preoperative imaging showed that the TBR of primary tumors was 4.12 ± 0.7 in the 5.0-μM group and 4.08 ± 2.0 in the 2.5-μM group. The mean fluorescence intensities (MFIs) of primary tumors and adjacent tissue were respectively 65.09 ± 21.8 a.u. and 18.23 ± 8.3 a.u. in the 5.0-μM group and 36.65 ± 30.9 a.u. versus 10.21 ± 10.6 a.u. in the 2.5-μM group.

**Fig. 2:**
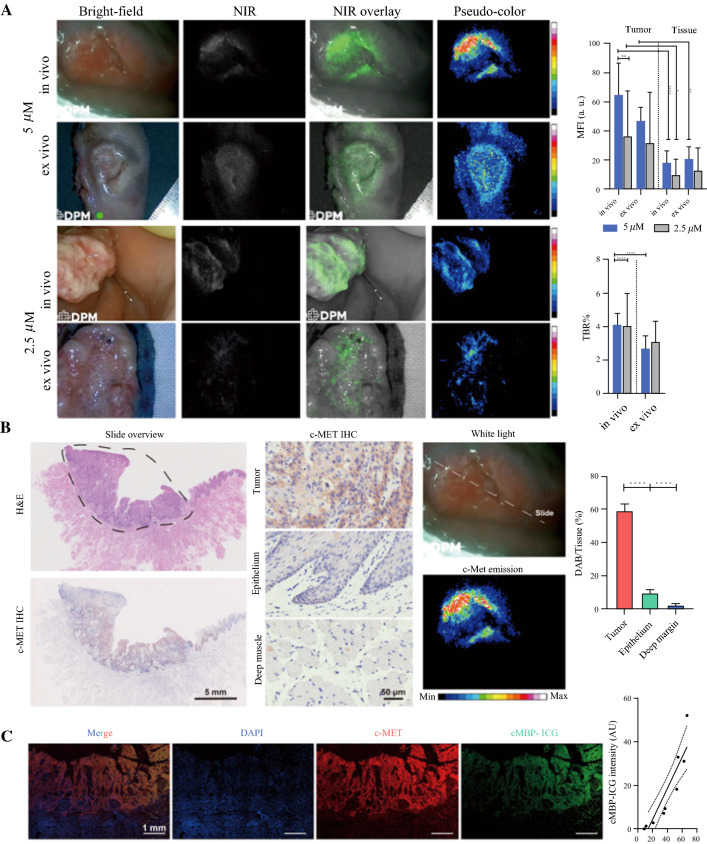
Analysis of cMBP-ICG dosage selection in vivo and ex vivo after fluorescence imaging, and correlation between c-MET expression and cMBP-ICG signal intensity. **A** Snapshots of two patients from different dosage groups. The MFI and TBR of each group and each application procedure (in vivo/ex vivo) were calculated following the same process. *p < 0.05, **p < 0.01, ***p < 0.001, ****p < 0.0001. Data are presented as mean ± SEM. **B** Representative c-MET immunohistochemistry images and hematoxylin and eosin images from a paraffin-embedded tumor section of a patient (*slide overview*) showing an area of head and neck squamous cell carcinoma (SCC) (*black circle*). Preoperative in vivo cMBP-ICG imaging of the patient showing the slide location in the whole tumor. **C** Immunofluorescence of the paraffin-embedded tumor shown in panel **B**. cMBP-ICG, c-MET-binding peptide-indocyanine green; MFI, mean fluorescence intensity; TBR, tumor-to-background ratio; SEM, standard error of the mean

The intraoperative imaging showed that the TBR of primary tumors was 2.71 ± 0.7 in the 5.0-μM group and 3.11 ± 1.2 in the 2.5-μM group. The MFIs of primary tumors and adjacent tissue were respectively 47.35 ± 9.0 a.u. and 21.14 ± 8.2 a.u. in the 5-μM group and 32.45 ± 34.2 a.u. versus 13.38 ± 15.2 a.u. in the 2.5-μM group (Fig. 2A).

Tissue samples from nine patients were available for c-MET expression analysis via IHC (excluding 1 non-tumor patient). Based on the pathologic assessment, c-MET IHC staining was analyzed and quantified in tumor, mucosal epithelium, and deep margin (i.e., healthy muscle tissue). The mean ratio of the c-MET area (marked by diaminobenzidine, DAB) over the total tumor tissue area was 57.3% ± 17.8%, which was significantly higher than in normal mucosal epithelium (8.4% ± 3.6%) or in the deep muscular margin (4.1% ± 2.2%), indicating high c-MET expression in the tumor (Fig. 2B; *p* < 0.0001 in both compared groups). The patients’ TBR, MFI, and DAB/tissue area were summarized in Fig. S3.

To determine the specificity and sensitivity of cMBP-ICG for neoplastic tissue, the MFI data from 60 tumor margin specimens were used to create an ROC curve. The area under the curve (AUC), the average positive predictive value (PPV), and the negative predictive value (NPV) are shown in Table 1.

**Table 1:** Sensitivity and specificity of fluorescence for tumor surface samples

Samples	Group 1	Group 2	Both groups	Surgeons
Concentration (μM)	5	2.5		
Sensitivity (%)	100	46	71	60
Specificity (%)	75	88	83	95
PPV	80	79	83	93
NPV	100	61	73	71
AUC	0.88	0.65	0.78	NA
95 % CI	0.88–1	0.53–0.833	0.68–0.88	NA

*PPV* Positive predictive value, *NPV* Negative predictive value, *AUC* Area under the curve, *CI* Confidence interval

Adjacent paraffin-embedded tumor sections were stained with c-MET/AF488, cMBP-ICG, and DAPI. Immunofluorescence (IF) images showed co-localization of c-MET/AF488 and cMBP-ICG with nearly identical tissue shapes. The correlation test showed that cMBP-ICG localization was significantly associated with c-MET localization (*R*^2^ = 0.8319; *p* < 0.01; Fig. 2C).

### cMBP-ICG Enables the Screening of Small Cancer Foci

**Fig. 3:**
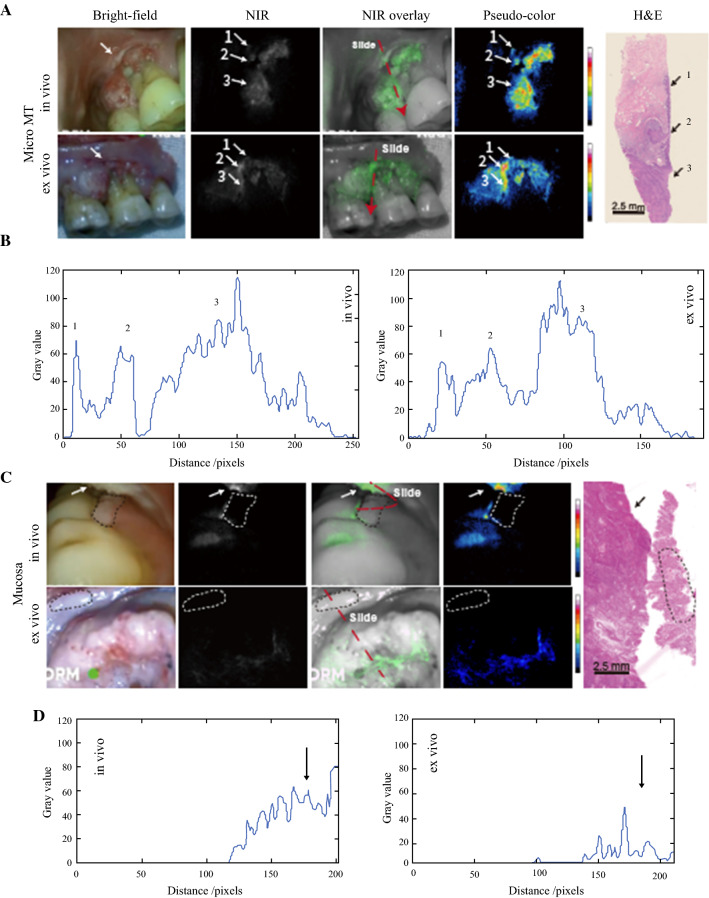
**A** Suspicious lesions discovered and **C** a nontumor lesion screened out during preoperative imaging procedure. Frozen sections were cut according to the real-time videos. The section locations (*red dashed line*) were ensured to meet the region of interest. **A** Severe dysplasia (*arrows 1 and 2*) detected 2 mm above the primary gum cancer area (*arrow 3*). **B** Two-dimensional (2D) fluorescence-intensity histogram illustrating the signal enhancement in the tumor area and the suspicious region. **C** A gum leukoplakia (*dashed circle*) adjacent to primary tongue cancer (*arrow*) that proved to be normal mucosa pathologically. **D** A 2D fluorescence-intensity histogram illustrating the signal enhancement in the tumor area. NIR, near infrared; H&E, hematoxylin and eosin

In one patient, a suspicious region was detected 2 mm above the primary gum cancer area during preoperative in vivo NIR fluorescence imaging. The attending surgeon expanded the surgical removal area accordingly. This suspicious micro-lesion also was visualized in intraoperative ex vivo NIR fluorescence imaging. An intraoperative frozen section was sliced with guidance from the ex vivo NIR fluorescence imaging, ensuring that the suspicious lesion was present in the frozen section. The pathology results verified this suspicious lesion as severe dysplasia of the epithelium, which had a high probability of cancerization (Fig. 3A). A two dimensional (2D) fluorescence intensity histogram showed the signal enhancement in the tumor area and the suspicious region (Fig. 3B).

The attending surgeon suspected a gum leukoplakia in a patient to be a local metastasis considering its anatomic proximity to the primary tongue tumor. However, the NIR fluorescence intensity of this area in preoperative in vivo imaging was not higher than that of the adjacent normal tissue. This finding did not affect the operation plan. Still, the NIR fluorescence intensity of this area in the excised specimen was not higher than in the adjacent normal tissue. The pathologic analysis of frozen section of this area showed normal mucosa (Fig. 3C). A 2D fluorescence intensity histogram showed the signal enhancement only in the tumor area (Fig. 3D).

### cMBP-ICG Shows Potential for Rapid Biopsy and Tumor Grading

**Fig. 4:**
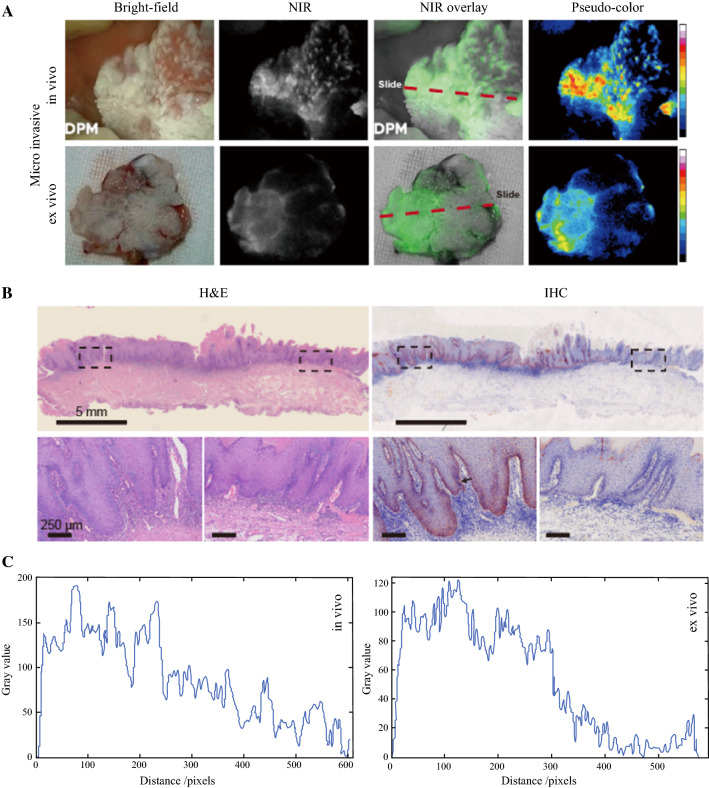
Heavily keratinized mucosa with small cancer foci in one patient. **A** Snapshots from preoperative (*upper row*) and intraoperative (*lower row*) imaging. **B** Hematoxylin and eosin (H&E) and immunohistochemistry (IHC) of the frozen sections. The lower row demonstrates enlarged views of the two areas framed in black in the upper row. The section locations (*red dashed line in panel A*) were ensured to meet the region of interest. **C** Two-dimensional (2D) fluorescence-intensity histogram illustrating the signal enhancement in the high c-Met expression area

The biopsy for one patient showed that one lesion of buccal mucosa was tumor negative. The attending physician speculated that it was a tumor and needed to be removed. High intensity of both in vivo and ex vivo NIR fluorescence suggested partial cancerization (Fig. 4A). Postoperative pathology results confirmed that the expression of c-MET in the high-fluorescence signal area was high, and that local small cancer foci were present in this area (Fig. 4B). A 2D fluorescence intensity histogram showed the signal enhancement in the high c-Met expression area (Fig. 4C).

**Fig. 5:**
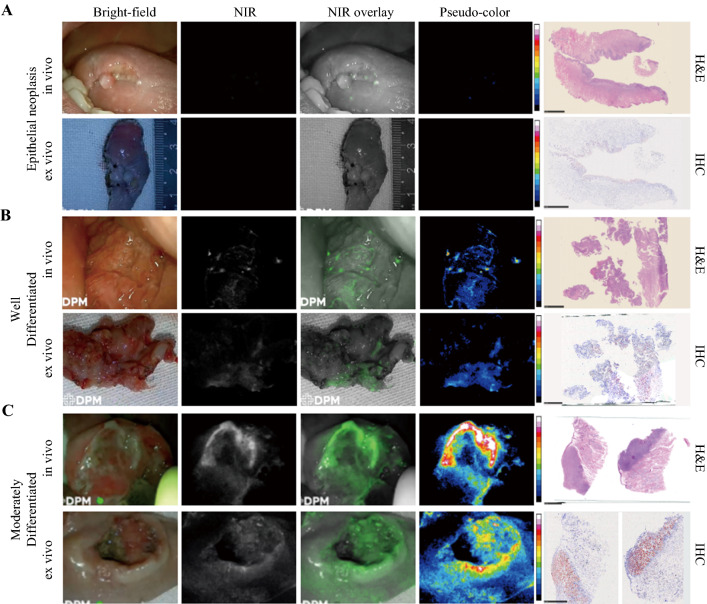
Comparison of epithelial neoplasia and two grades of head and neck squamous cell carcinoma (SCC). **A** Epithelial neoplasia showing low fluorescence intensity (FI) in both in vivo and ex vivo imaging. The frozen section shows low c-MET expression of this lesion. **B** Highly differentiated head and neck SCC showing slightly increased FI compared with panel A. The frozen section shows about 25% of the c-MET expression area fraction. **C** Moderately differentiated head and neck SCC showing high FI in in vivo and ex vivo imaging. The frozen section shows about 90% of the c-MET expression area fraction

The 10 cases in this study included intraepithelial neoplasia (*n* = 1) (Fig. 5A), well-differentiated oral SCC (*n* = 2), moderately well-differentiated oral SCC (low grade) (*n* = 6) (Fig. 5B), and moderately differentiated oral SCC (intermediate grade) (*n* = 1) (Fig. 5C). The MFI and TBR of the moderately well-differentiated oral SCC and the moderately differentiated ORAL SCC were analyzed by unpaired *t* test (Fig. S4). There were significant differences in all the outcomes.

The topical usage of cMBP-ICG at a relatively high concentration did not result in any grade 1 or higher adverse events for the patients with oral SCC.

## Discussion

The detection of mucosal extension and skip lesion is essential for patients with oral SCC for complete resection and minimized functional impairment. Lugol’s iodine staining is a traditional method for imaging and screening of cancer, primarily in esophageal cancer and cervical cancer. Although this technique is sensitive, its specificity is relatively low, and it is time-consuming. Lugol stain also leads to esophageal spasms and sometimes pain in Lugol chromoendoscopy, which might be more insufferable for oral cancer patients. Moreover, it is unavailable for non-keratinized mucosa detection and not related to pathologic grading of oral SCC.^18,19^

For superficial oral lesions using tumor-targeting fluorescent agents, NIR fluorescence-guided surgery is a promising alternative. The advantages of NIR ligands include greater penetration depth and lower autofluorescence than fluorophores emitting in the visible-wavelength range.^7,8,20,21^ As reported previously, more than 60–80% of oral SCCs have shown c-Met protein overexpression.^22–24^ Therefore, c-Met could serve as a promising biomarker for diagnosis and imaging of oral SCC.

Based on our previous work,^16^ cMBP peptide was selected as the targeting ligand because of its high docking affinity to c-Met and known application in tumor therapy^25^ and imaging.^26,27^ This homing peptide may offer many properties including high tumor penetration, low immunogenicity, and cheap synthesis.^28^ Indocyanine green was chosen as the imaging agent due to its biomedical safeness and high signal-to-background ratio.^29^

In this study, a coating concentration of 5.0-μM displayed better imaging contrast in a dark room during preoperative in vivo experiment. In contrast, under intraoperative ex vivo conditions, surgical lighting background in the operation theater caused higher background noise to sensors of the camera, and the concentration of 2.5-μM ensured a higher TBR. Regarding visual perception of real-time fluorescence imaging, the tumor area could be visualized more clearly in 5.0-μM group. High TBR from the 2.5-μM group beneficated from low background noise, but it did not effectively help to discriminate tumor regions. Therefore, MFI may be a more suitable index to referee tumor fluorescence imaging effectiveness. The ROC results in this study demonstrated that the 5.0-μM group had a higher AUC. The PPV and NPV also were higher in the 5.0-μM group, agreeing with the visual effect.

Due to the “areal cancerization” feature of oral SCC,^4^ microscopic cancer foci or premalignant lesions may discretely occur around the primary cancer area. This characteristic is a key underlying cause for the high relapse rate of oral SCC. We believe that the first patient (Fig. 3A, whose occult lesion was detected by fluorescence imaging) benefited from the approach used in this study. Our NIR fluorescence imaging method was proven capable of identifying and locating such small cancerous foci or precancerous lesions. The attending surgeon can revise surgical procedure accordingly to suppress the probability of cancer relapse.

Oral SCC can invade various mucosa spaces through mucosa folds and tunnels.

For the second patient (Fig. 3B), the suspicious leukoplakia was located in an area highly suggestive of a metastasis. Conservatively, surgeons are inclined to excise such lesions. However, oral environments of patients with oral cancer usually are unhealthy, exhibiting mucocutaneous striatal abnormalities without pathologic significance. Excessive excision may unnecessarily impair the physiologic function. Near-infrared fluorescence imaging may assist the conventional technique in differentiating cancer foci for long-term follow-up of mucosal lesions and in preventing overtreatment.

Biopsy is not very effective in detecting early-stage carcinoma *in situ* given that the cancerized area may be too small and the biopsy puncture area may not cover the cancerized area. The aberrant activation and overexpression of c-MET is a very early event in the development of squamous cell carcinoma.^30^ In the current study, topical application of c-MET-targeting fluorescence dye was superior in detecting the early-stage cancer. Expression of C-MET varied with the severity of epithelial dysplasia. Specifically, the level of c-MET expression descended from carcinoma *in situ* via highly atypical hyperplasia to normal mucosa. For the patient mentioned in the third RESULTS section, the observed NIR fluorescence intensity agreed well with this trend, reflecting c-MET expression. Therefore, topical application of cMBP-ICG can be used to guide biopsy sampling and may shift the detection of oral SCC to an earlier time point.

This study had many strengths. First, topically applied real-time imaging agents are well-suited for the study of head and neck cancer.^14^ Given that oral SCC develops from the mucosal epithelium in the oral cavity, pharynx, and larynx^31^ and is present on the oral and oropharyngeal surfaces, gargling may be an excellent choice for noninvasive and convenient prompt investigation. Namely, the mouth-washing topical application of the targeting imaging agents may be an optimal approach given that mouthwash covers the entire oral mucosa and directly screens out suspicious small cancer foci without restriction from the other tissues.

Second, the topical application significantly reduced the imaging time of the NIR fluorescence probes (within minutes).^13,14,32^ The fast renal clearance of peptide-based targeting agents’^28^ is not seen as a drawback, but rather as a benefit from the perspective of medication safety.

The relatively small sample size limited this research. The tumor tissues resided sub-surface. NIR fluorescence imaging was conducted at surfaces of ROI, while imaging information of cross-section of tumor tissues was not obtained. Relevant study regarding in-depth imaging is ongoing. The NIR imaging obtained in this study cannot be compared with that obtained via intravenous injection. More studies on surface application of NIR contrast agents may address the biases of single-center clinical trials.

## Conclusion

In conclusion, the topical application of cMBP-ICG showed good biosafety and specificity for oral SCC. Although the current clinical trial was a pilot study, it suggested the remarkable potential of cMBP-ICG for delineating tumor margins, showing concealed minor tumor foci and micro metastasis and indexing oral SCC differentiation grades. Also, as a quick, convenient, and economical method for detecting oral SCC, c-MBP-ICG could be potentially used in the near future for long-term follow-up evaluation of leukoplakia, guidance of biopsy and surgical procedure, and postoperative evaluation of oral SCC patients.

## Supplementary Information

Below is the link to the electronic supplementary material.

Supplementary file1 (DOCX 835 KB)
