# Supplementary material for: A c-MET-Targeted Topical Fluorescent Probe cMBP-ICG Improves Oral Squamous Cell Carcinoma Detection in Humans
Source: Ann Surg Oncol. 2022 Oct 2;30(1):641–51. doi: 10.1245/s10434-022-12532-x (PMC9726820; doi:10.1245/s10434-022-12532-x)
Supplement: Supplementary file 1 — Supplementary file1 (DOCX 835 KB) [file 10434_2022_12532_MOESM1_ESM.docx]

Supporting Information

**A c-MET-Targeted Topical Fluorescent Probe cMBP-ICG Improves Oral Squamous Cell Carcinoma Detection in Humans**

*Jingbo Wang, Siyi Li, Kun Wang, Ling Zhu, Lin Yang, Yunjing Zhu, Zhen Zhang, Longwei Hu, Yuan Yuan, Qi Fan, Jiliang Ren, Gongxin Yang, Weilong Ding, Xiaoyu Zhou, Junqi Cui, Chunye Zhang, Ying Yuan*, Ruimin Huang*, Jie Tian*, Xiaofeng Tao**


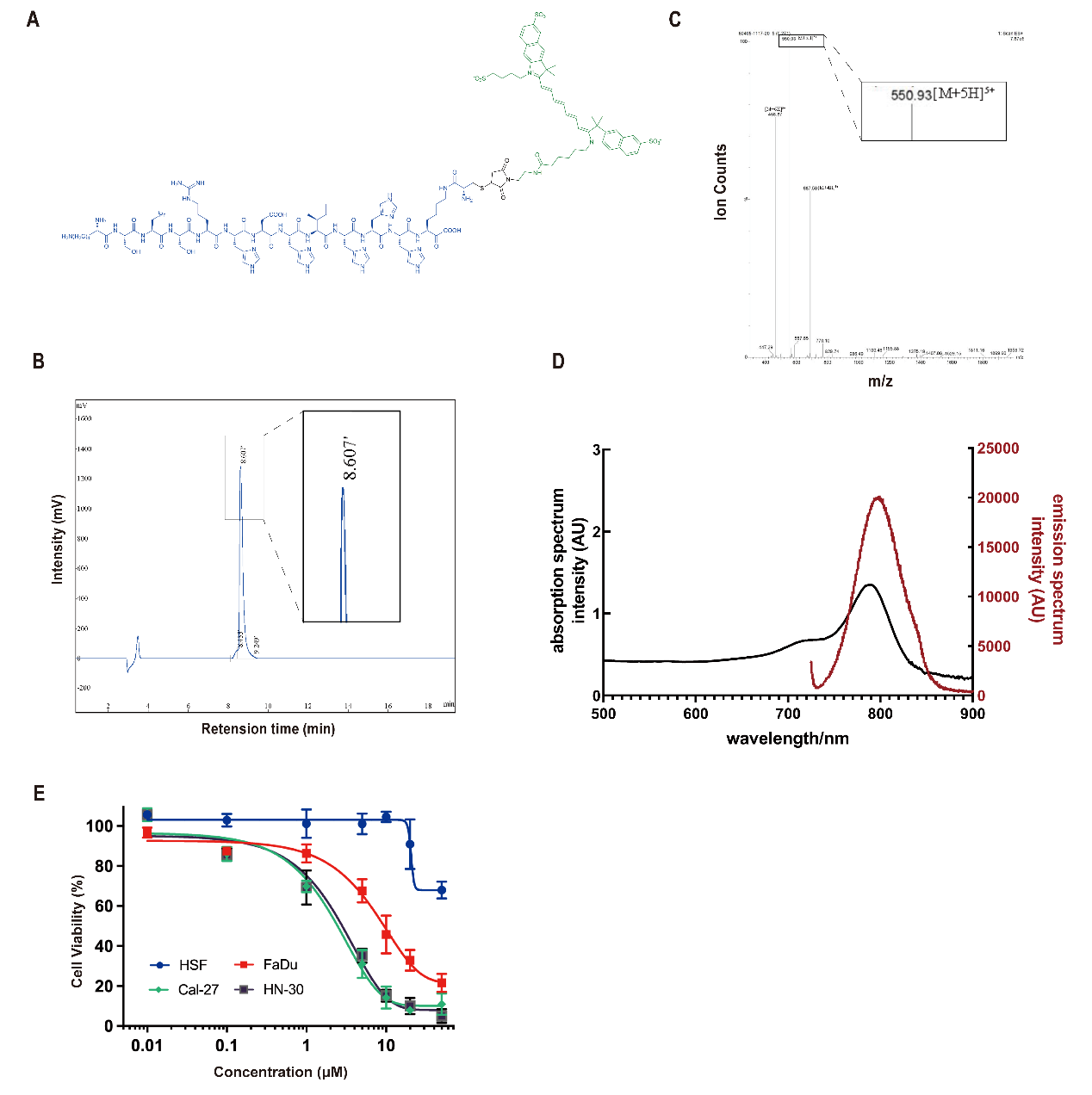


**Supplementary Figure S1.** Structure and biophysical properties of cMBP-ICG. (A) Molecular structure of cMBP-ICG (blue = c-MET binding domain; green = ICG fluorescence dye moiety). (B) The retention time of cMBP-ICG was 8.61 minutes at 5%–95% linear gradient of acetonitrile (0.5% TFA) in water (0.1% TFA) in 20 min. (C) The identity of cMBP-ICG confirmed us (+) m/z = 550.93 [M+5F] ^5+^ (D) The absorption/emission spectrum of cMBP-ICG was 789/803nm. (E) In vitro cytotoxicity assessment of cMBP-ICG against HNSCC cell lines and a control keratin epithelial cell line after 48 h of treatment. The IC50 of cMBP-ICG was 3.38 μM.


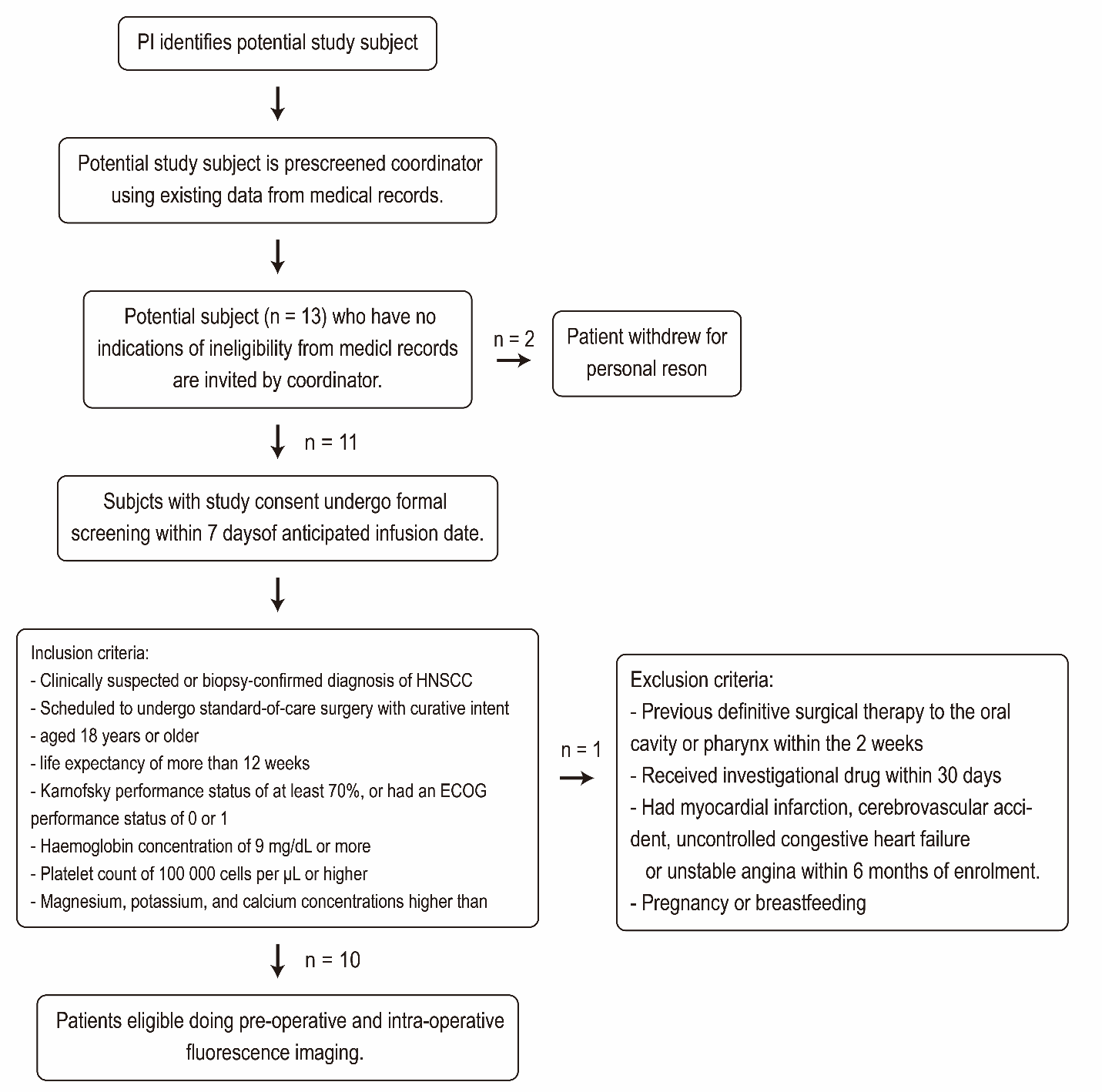


**Supplementary Figure S2.** Flowchart for the study.


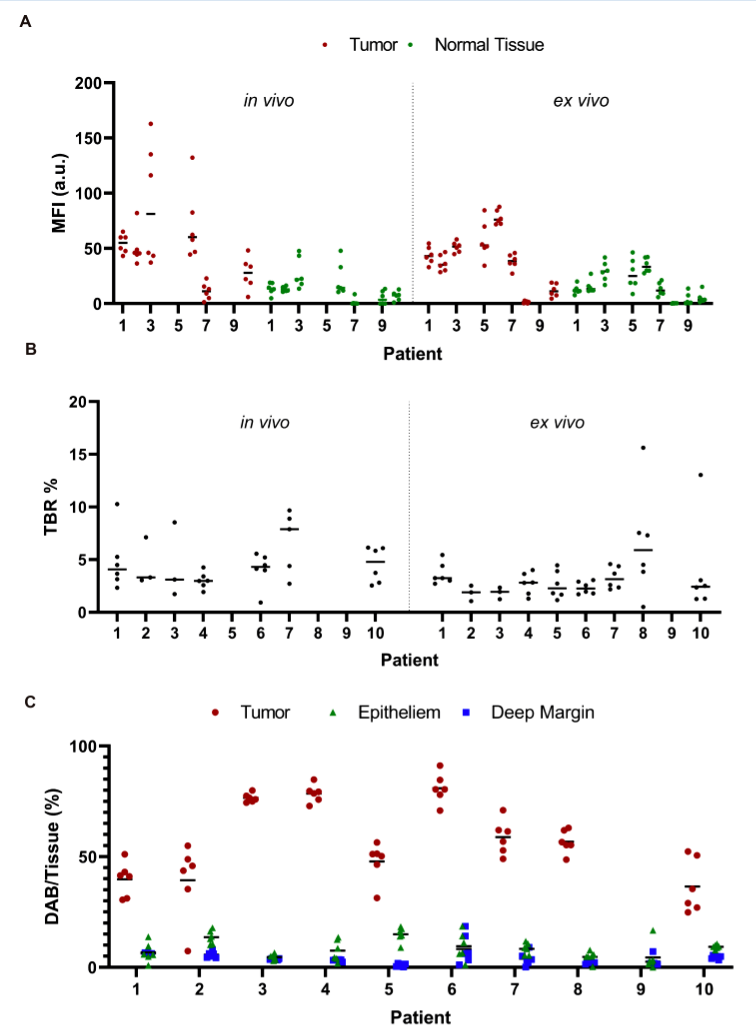


**Supplementary Figure S3**. Quantification of c-MET expression in immunohistochemistry, immunofluorescence samples, and pre-/intraoperative cMBP-ICG florescence (n = 10 patients). MFI (A), TBR (B), and DAB/Tissue area (C) were measured with Pearson correlation. In vivo imaging was not performed for patient 5 and patient 8 due to limitation of mouth opening. Patient 9 was a non-tumor patient, so it’s MFI was not included in TBR statistics.


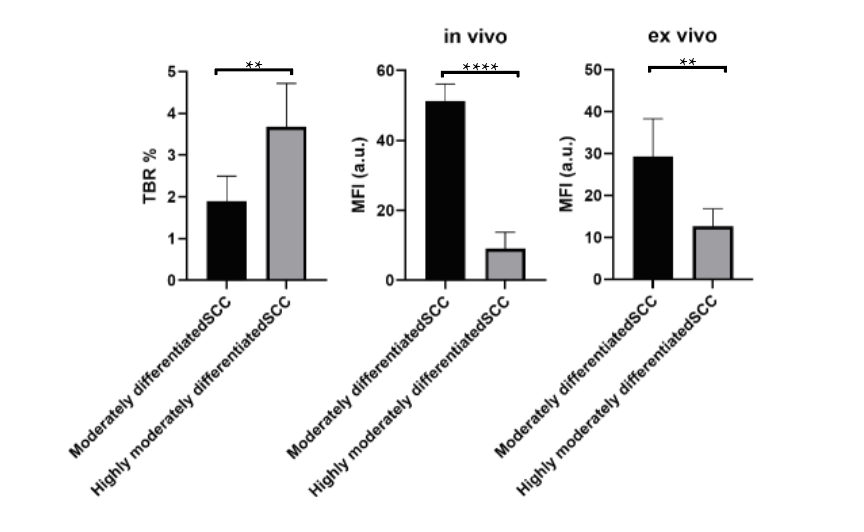


**Supplementary Figure S4**. Unpaired *t* test for TBR and MFI of highly moderately differentiated and moderately differentiated HNSCC.

**Supplementary Table S1.** Demographic and clinical information for 10 cases applied in clinical trials

|  | Patient ID | Age | Gender | Surgical procedure | Tumour site | Histopathology diagnosis | Pathology stage | size (cm) | Previous  chemotherapy | Previous radiotherapy | |
| --- | --- | --- | --- | --- | --- | --- | --- | --- | --- | --- | --- |
| 5 μM | 1 | 50 | male | Extended local excision | left lingual margin | Highly moderately differentiated SCC | T1N0M0 | 1.5 | No | | No |
|  | 2 | 67 | male | Extended local excision | right gums | Highly moderately differentiated SCC | T1N0M0 | 2 | No | | No |
|  | 3 | 64 | male | Combined radical OP of jaw and neck | left lingual margin | Moderately differentiated SCC | T1N0M0 | 2 | No | | No |
|  | 4 | 33 | male | Extended local excision+  Ipsilateral lymph node dissection | left Maxilla | Highly moderately differentiated SCC | T3N2M0 | 5 | No | | No |
|  | 5 | 62 | male | Extended local excision | right gums | Highly moderately differentiated SCC | Relapse | 3 | 3 cycles | | No |
| 2.5 μM | 6 | 56 | male | Extended local excision | left Buccal mucosa | Highly moderated SCC | TisN0M0 | 6 | No | | No |
|  | 7 | 55 | male | Combined radical OP of jaw and neck | right gums | Highly moderately differentiated SCC | Relapse | 3 | No | | No |
|  | 8 | 61 | male | Extended local excision+  Ipsilateral lymph node dissection | left lingual margin | Highly moderately differentiated SCC | T3N2M0 | 5 | cex 1 cycle | | No |
|  | 9 | 82 | male | Extended local excision | right dorsal tongue | Epithelial neoplasia | / | 3.5 | No | | No |
|  | 10 | 64 | female | Extended local excision | left lingual margin | Highly moderated SCC | T3N0M0 | 1.5 | No | | No |
